# Genetic structure among greater white‐fronted goose populations of the Pacific Flyway

**DOI:** 10.1002/ece3.2934

**Published:** 2017-03-22

**Authors:** Craig R. Ely, Robert E. Wilson, Sandra L. Talbot

**Affiliations:** ^1^ U.S. Geological SurveyAlaska Science CenterAnchorageAKUSA

**Keywords:** *Anser albifrons*, gene flow, pairing behavior, phylogeography, population structuring

## Abstract

An understanding of the genetic structure of populations in the wild is essential for long‐term conservation and stewardship in the face of environmental change. Knowledge of the present‐day distribution of genetic lineages (phylogeography) of a species is especially important for organisms that are exploited or utilize habitats that may be jeopardized by human intervention, including climate change. Here, we describe mitochondrial (mtDNA) and nuclear genetic (microsatellite) diversity among three populations of a migratory bird, the greater white‐fronted goose (*Anser albifrons*), which breeds discontinuously in western and southwestern Alaska and winters in the Pacific Flyway of North America. Significant genetic structure was evident at both marker types. All three populations were differentiated for mtDNA, whereas microsatellite analysis only differentiated geese from the Cook Inlet Basin. In sexual reproducing species, nonrandom mate selection, when occurring in concert with fine‐scale resource partitioning, can lead to phenotypic and genetic divergence as we observed in our study. If mate selection does not occur at the time of reproduction, which is not uncommon in long‐lived organisms, then mechanisms influencing the true availability of potential mates may be obscured, and the degree of genetic and phenotypic diversity may appear incongruous with presumed patterns of gene flow. Previous investigations revealed population‐specific behavioral, temporal, and spatial mechanisms that likely influence the amount of gene flow measured among greater white‐fronted goose populations. The degree of observed genetic structuring aligns well with our current understanding of population differences pertaining to seasonal movements, social structure, pairing behavior, and resource partitioning.

## 1 Introduction

A firm understanding of population genetic structure is a cornerstone of informed conservation management for wildlife species (Avise, 2000; Palsbøll, Bérubé, & Allendorf, 2006). This is especially true of widely distributed species that may be composed of populations connected by varying degrees of ecological or evolutionary dispersal (Pruett et al., 2008). Ecological dispersal (natal and breeding dispersal) can be measured directly through observation and marking studies, and evolutionary dispersal (gene flow) can be measured via genetic investigations. While preferably, both measures of within‐species connectivity should be used (Alvarado, Fuller, & Smith, 2014; Lecomte, Gauthier, Giroux, Milot, & Bernatchez, 2009), such measurements can be complex and logistically difficult. This is particularly true for migratory birds, given their often extensive population ranges and tremendous dispersal potential. Overcoming such difficulties is paramount for species breeding in the Arctic, as habitats there are changing in relation to rapidly warming northern climates (Pearson et al., 2013), and species ranges and migratory patterns are predicted to change over time (Sorte & Jetz, 2010). Securing baseline information on population structure for hunter‐harvested Arctic species, such as northern waterfowl, is of added importance given the potential for overharvesting, which can reduce genetic diversity through elimination of unique genetic units (Allendorf & Hard, 2009).

**Figure 1:**
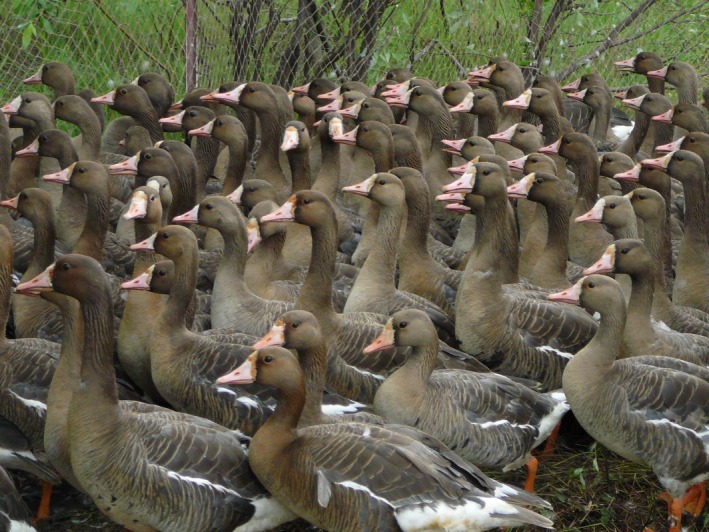
Mixed molting group of greater white‐fronted geese (*Anser albifrons*) near the Innoko River, Alaska, USA

**Figure 2:**
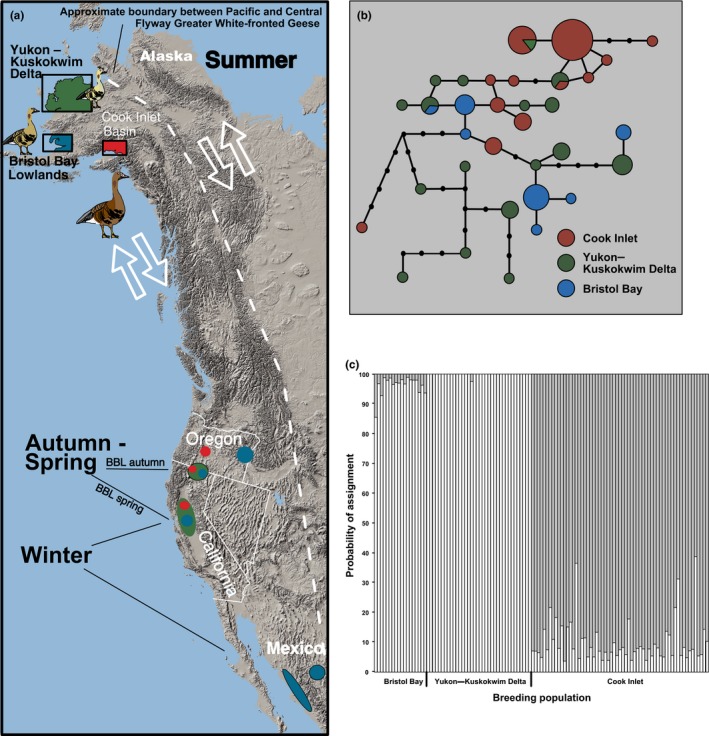
(a) Distribution of greater white‐fronted geese (*Anser albifrons*) in the Pacific Flyway, showing breeding, migration, and wintering locations of geese from the three primary breeding areas (after Ely, 2008). (b) Unrooted 95% parsimony network showing the relationships of 33 haplotypes from 365 base pair sequences of the mtDNA control region from greater white‐fronted geese from three Pacific Flyway populations. The size of the circles is proportionate to number of individuals. Small black circles represent intermediate haplotypes that were not sampled. (c) Output of STRUCTURE analysis using LOCIPRIOR (*r* < 1) for greater white‐fronted geese from three different breeding areas in Alaska. Sampling location was used as a prior (see Section 2)

We therefore undertook a population genetic study of the greater white‐fronted goose (*Anser albifrons*) in the Pacific Flyway of North America (Figure 1). The greater white‐fronted goose, a migratory species with a Holarctic distribution, is harvested by sport and subsistence hunters throughout much of its range, especially in North America. In the Pacific Flyway, white‐fronted geese nest in three areas of Alaska: the Yukon–Kuskokwim Delta (YKD) of western Alaska, the Bristol Bay Lowlands (BBL) in southwestern Alaska, and the Cook Inlet Basin (CIB) of south central Alaska (Figure 2a). Previous investigations of white‐fronted geese in the Pacific Flyway revealed differences among geese sampled from these three breeding locales with respect to morphology (Ely et al., 2005; Orthmeyer, Takekawa, Ely, Wege, & Newton, 1995), distribution (Ely & Takekawa, 1996), and timing of migration and reproduction (Ely, 2008; Ely & Takekawa, 1996), such that the three locales are considered to comprise discrete nesting populations. The populations are allopatric during the summer nesting season, but overlap in distribution during the nonbreeding season (Ely & Takekawa, 1996; Ely, 2008; Figure 2a). The populations also differ with respect to timing of migration (BBL geese migrate earlier in spring and fall than the other two populations) and nesting (BBL and CIB geese nest earlier than YKD geese; Ely & Takekawa, 1996; Ely et al., 2005, 2007). Due to concerns mentioned above, we assessed the population genetic structure of geese from these three nesting locales by using genotypic data from nuclear microsatellite loci and sequence data from mitochondrial DNA (mtDNA) control region.

Studies of other Arctic nesting geese in North America have found varying levels of within‐species population differentiation, including instances of deep divergences consistent with species‐level differences, and hybridization. For example, fragment data from nuclear microsatellites showed substantial population structuring in Canada goose (*Branta canadensis*) populations nesting in high latitudes in Alaska and western North America, but also found deep cleavages in mtDNA haplotypes between what were previously called the large‐ and small‐bodied Canada goose lineages, findings that contributed to taxonomic revision of the group into two species, the Canada goose and the cackling goose (*B. hutchnisii*), respectively (Banks et al., 2004; Paxinos et al., 2002; Scribner et al., 2003). Leafloor, Moore, and Scribner (2013) demonstrated historical hybridization between cackling and Canada geese in high‐latitude Canada. Avise, Alisauskas, Nelson, and Ankney (1992) found two major clades of mtDNA in the snow goose (*Chen caerulescens*), and Quinn (1992) found that within one clade, mtDNA was concordant with geographic location, while no such concordance was found within the other clade. Ross’ goose, *C. rossii*, is considered a sister species of the snow goose and the two species are thought to frequently hybridize (Weckstein, Afton, Zink, & Alisauskas, 2002). While within‐flyway spatial genetic structure is evident for both the Canada and cackling goose (Scribner et al., 2003), genetic structure in snow geese did not correlate with flyway (Shorey, Scribner, Kanefsky, Samuel, & Libants, 2011).

Although greater white‐fronted geese from the three locales sampled in our study occupy a single migratory flyway, we nevertheless anticipated some degree of population genetic differentiation given that mtDNA is maternally inherited and most species of waterfowl exhibit female natal philopatry (Greenwood, 1980). Evidence of population genetic structuring also seemed likely as the three populations vary in body size (Ely et al., 2005; Orthmeyer et al., 1995), with the distinctly larger structural size of Tule geese (CIB population) likely contributing to resource partitioning (CIB geese are adapted to feed on aquatic marsh plants; Ely, 2008), and reproductive isolation through sexual imprinting mechanisms. We also expected gene flow among the populations to be lowest between CIB and the other populations, given the likelihood that the populations are allopatric during the time of mate selection. If pair formation occurs during spring or summer, then despite autumn and winter sympatry, gene flow among populations would be impeded, with CIB geese likely being the most isolated. Genetic structure may also be evident if mate choice is affected by population differences in timing of migration and nesting; this is especially likely in capital breeders such as geese, which rely on rapid accumulation of reserves for breeding and migration during a very short period in spring (Ely & Takekawa, 1996). Here, we present the results from the first population genetic study of greater white‐fronted geese, explore possible mechanisms leading to observed patterns, and examine how our findings differ from studies of ducks and species of geese with different life history features.

## 2 Methods

### 2.1 Sample collection

Blood, feather, or eggshell membranes were collected from greater white‐fronted geese at three different locales in Alaska within the Pacific Flyway (BBL, *n *= 18; YKD, *n *= 36; and CIB, *n *= 61; Figure 2a) between 1989 and 2006. Blood samples were stored in blood preservation buffer (Longmire et al., 1988) and feather and egg membranes were placed in envelopes and stored at room temperature. All samples are archived at the Molecular Ecology Laboratory, Alaska Science Centre (ASC), U.S. Geological Survey (USGS) in Anchorage, Alaska, where detailed sample information is also available (http://doi.org/10.5066/F71G0JGN). Geese were captured and banded under the auspices of the U.S. Fish and Wildlife Service Region 7, and the USGS, Alaska Science Centre, under Federal Permit # MB789758.

### 2.2 Laboratory techniques

Genomic DNA was extracted from blood, muscle, feather, or eggshell membranes using a “salting out” procedure (Medrano, Aasen, & Sharrow, 1990) with modifications for blood and muscle (Sonsthagen, Talbot, & White, 2004) and feathers and eggshell membranes (Talbot et al., 2011). Genomic DNA concentrations were quantified using fluorometry and diluted to 50 ng/ml working solutions. Initially 12 individuals were screened at 26 loci known to be variable in other waterfowl species. Eight presumably unlinked polymorphic loci with dinucleotide repeat motifs and in Hardy–Weinberg equilibrium were selected for further analysis: BCA6, BCA9, BCA11 (Buchholz, Pearce, Pierson, & Scribner, 1998), CRG (Wilson, Gust, Petersen, & Talbot, 2016), Aaμ1 (Fields & Scribner, 1997), OXY13 (Muñoz‐Fuentes, Gyllenstrand, Negro, Green, & Vila, 2005), TSP1.20.09, and TSP.1.20.46 (John, Ransler, Quinn, & Oyler‐Mccance, 2006). Polymerase chain reaction (PCR) amplification and electrophoresis followed standard protocols (Sonsthagen et al., 2004). Ten percent of the samples were amplified and genotyped in duplicate for the eight microsatellite loci for quality control. There were no differences between initial and duplicate amplifications and genotypes. Microsatellite genotype data are accessioned at the USGS, ASC data repository (https://doi.org/10.5066/F71G0JGN).

We also amplified a portion of domain I and domain II of the mitochondrial DNA (mtDNA) control region using the primer pair WFGL1M (5′–ACTAACCGCGAACTCCCAAA–3′) and H542 (Sorenson & Fleischer, 1996), yielding a 365 base pair sequence product for all individuals. Ruokonen, Kvist, and Lumme (2000) showed that approximately half of the variable sites in lesser white‐fronted goose (*A. erythopus*) in the mtDNA control region are within control region domain I. Using the methods in Lanctot et al. (1999) and using previously published sequences, we verified the amplified fragment was mitochondrial in origin. PCR amplifications, cycle‐sequencing protocols, and postsequencing processing followed Sonsthagen et al. (2004). All sequences have been submitted to GenBank (accession numbers: KY704180‐KY704263).

### 2.3 Genetic diversity

We calculated allelic richness, the inbreeding coefficient (*F*_IS_), observed and expected heterozygosities, Hardy–Weinberg equilibrium (HWE), and linkage disequilibrium (LD) for each microsatellite locus and population in FSTAT ver. 2.9.3 (Goudet, 1995). We used ARLEQUIN ver. 3.5.1.2 (Excoffier & Lischer, 2010) to estimate haplotype diversity (*h*—the probability that two randomly chosen haplotypes are different), and nucleotide diversity (π—the average number of nucleotide differences per site between two randomly chosen DNA sequences) for the mtDNA control region sequence data. An unrooted phylogenetic tree for mtDNA control region was constructed in NETWORK 4.6.1.3 using the median joining method (Bandelt, Forster, & Röhl, 1999), to illustrate possible reticulations in the gene tree as a result of homoplasy or recombination.

### 2.4 Population subdivision

The degree of subdivision among breeding areas was assessed by calculating pairwise *F*_ST_ and *R*_ST_ for microsatellite data, and *Φ*_ST_ for mtDNA data using ARLEQUIN, and adjusting for multiple comparisons using Bonferroni correction (α = 0.05) for microsatellite data. For sequence data, pairwise Φ_ST_ was calculated using the best‐fit nucleotide substitution model, as identified in MODELTEST 3.06 (Posada & Crandall, 1998) under Akaike's information criterion (AIC; Akaike, 1974). Because the upper possible *F*_ST_ value for a set of microsatellite loci is usually <1.0 (Hedrick & Goodnight, 2005), we used RECODEDATA, version 1.0 (Meirmans, 2006), to calculate the uppermost limit of *F*_ST_ for a given data set.

We also used a Bayesian‐clustering program, STRUCTURE 2.2.3 (Pritchard, Stephens, & Donnelly, 2000), to determine the level of population structure in the autosomal microsatellite data set without providing a priori information on the geographic origin of the individuals. If no structure was observed, we used the LOCPRIOR option as this model is able to detect population structure in data sets with a weak signal of structure not detectable under standard models (Hubisz, Falush, Stephens, & Pritchard, 2009). The analysis was run for *K *= 1–10, where *K* is the number of populations, using an admixture model with 100,000 burn‐in iterations and 1,000,000 Markov chain Monte Carlo (MCMC) iterations. The analyses were repeated ten times for each *K* to ensure consistency across runs. We used the ∆*K* method of Evanno, Regnaut, and Goudet (2005) and evaluated the estimate of the posterior probability of the data given *K*, Ln *P*(*D*), to determine the most likely number of groups at the uppermost level of population structure.

### 2.5 Historical population demography

Evidence for fluctuations in historical population demography was evaluated for eight microsatellite loci using BOTTLENECK 1.2.02 (Cornuet & Luikart, 1996). BOTTLENECK compares the number of alleles and gene diversity at polymorphic loci under the infinite allele model (IAM; Maruyama & Fuerst, 1985), stepwise mutation model (SMM; Ohta & Kimura, 1973), and two‐phased model of mutation (TPM; Di Rienzo et al., 1994). Parameters for the TPM were set at 79% SMM with a variance of 9% (Garza & Williamson, 2001; Piry, Luikart, & Cornuet, 1999), with 1,000 simulations performed for each population. Significance was assessed using a Wilcoxon sign‐rank test, which determines whether the average of standardized differences between observed and expected heterozygosities is significantly different from zero (Cornuet & Luikart, 1996). Significant heterozygote deficiency relative to the number of alleles indicates recent population growth, whereas heterozygote excess relative to the number of alleles indicates a recent population bottleneck (Cornuet & Luikart, 1996). BOTTLENECK compares heterozygote deficiency and excess relative to number of alleles, not to HWE expectation (Cornuet & Luikart, 1996).

Demographic histories based on mtDNA sequence data were evaluated using two approaches: standard qualitative test statistics and coalescent‐based estimations. To test for genetic signatures of recent effective population size changes, we calculated Fu's *F*_s_ (Fu, 1997) and Tajima's *D* (Tajima, 1989) on the basis of the site‐frequency spectrum of segregating sites. Negative values of Tajima's *D* or Fu's *F*_s_ result when there is an excess of low‐frequency polymorphisms, which can result from rapid population expansion or selective sweep acting on linked polymorphisms. Conversely, a positive value for either test statistic can be indicative of a population decline. We used a coalescent model in LAMARC 2.1.8 (Kuhner, 2006) to calculate the population‐growth‐rate parameter (*g*) for mtDNA from each population independently. We used a Bayesian analysis with 1,000,000 recorded genealogies sampled every 50 steps, with a burn‐in of 100,000 (10%) genealogies. Priors were flat with the upper limit for growth set to 15,000.

### 2.6 Estimation of gene flow

Estimates of contemporary (short term), recent (long term), and historical gene flow among sampled sites were calculated using two different models: assignment methodology in BayesAss v3.0 (Wilson & Rannala, 2003) and a steady‐state two‐island model of population differentiation in MIGRATE (Beerli & Felsenstein, 1999, 2001). These programs also differ in the temporal scale in which they estimate migration rates. BayesAss estimates migration (*m*) over the last several generations using allelic frequency data and does not assume that populations are in migration–drift or HWE. MIGRATE, in contrast, estimates recent (<10,000 years for microsatellites) and historical (mtDNA) migration rates (*N*_e_ *m* and *N*_f_ *m*), respectively (Wang, 2010), where effective population sizes (θ) are based on the coalescence and populations are assumed in migration–drift equilibrium. Therefore, gene flow estimates are averaged over the past *n* generations, where *n* equals the number of generations the populations have been at equilibrium (Beerli & Felsenstein, 1999, 2001). In addition, the primary mutation mechanism between microsatellite repeat units and nucleotide substitutions in mtDNA differ (Hancock, 1999); mtDNA loci have a slower rate of mutation (4.8 × 10^−8^ substitutions/site/year; Peters, Gretes, & Omland, 2005) and thus a deeper coalescence than nuclear microsatellite loci (mutation rate 10^−2^–10^−5^; Hancock, 1999). Therefore, results based on mtDNA sequence data provide a relatively more historical genetic signature than microsatellite fragment data.

BayesAss was initially run with the default delta values for allelic frequency (*P*), migration rate (*m*), and inbreeding (*F*). Subsequent runs incorporated different delta values to ensure that proposed changes between chains at the end of the run were between 20% and 40% of the total chain length to maximize log likelihood values and ensure the most accurate estimates (Wilson & Rannala, 2003). Final delta values used were Δ*A* = 0.40 (38% acceptance rate), Δ*m* = 0.15 (37%), and Δ*F *= 0.80 (33%). We performed five independent runs (20 million iterations, 2 million burn‐in, and sampling frequency of 2,000) with different random seeds to ensure convergence across runs. Convergence was also assessed by examining the trace file program Tracer v1.6 to ensure proper mixing of parameters (Rambaut, Suchard, Xie, & Drummond, 2014).

MIGRATE was run with a full migration model, θ (4*N*_e_μ, composite measure of effective population size and mutation rate), and all pairwise migration parameters were estimated individually from the data. Gene flow was estimated using a maximum‐likelihood search parameters; 10 short chains (5,000 trees used out of 1,500,000 sampled), ten long chains (15,000 trees used out of 5,250,000 sampled), and five static heated chains (1.0, 1.33, 2.0, 4.0, and 1,000,000; swapping interval = 1). Full models were run ten times to ensure the convergence of parameter estimates.

## 3 Results

### 3.1 Effects of sampling time periods

Because genetic samples from CIB and YKD were sampled over a number of years, we used a chi‐square test to determine if the allelic frequencies differed across sampling periods (Raymond & Rousset, 1995; Rousset, 2008). The YKD samples were divided into an early time period (1989, 1990, and 1992; *n *= 30) and a late time period (2006; *n *= 6). CIB samples were also broken down into early (1995, 1997; *n *= 36) and late (2000; *n *= 25) collections. We found no differences across time for either YKD geese (χ² = 17.25, *df *= 16, *p *= .37), or CIB geese (χ² = 12.95, *df *= 16, *p *= .67). This result was not unexpected, as geese have a long generation time (5–8 years; Dillingham, 2010) and a long life span (>6 years), so our main sampling period (1989–2000) spanned only a few generations, which would reduce the potential effects of genetic drift on allelic and haplotype frequencies.

### 3.2 Genetic diversity and population structure

Thirty‐three unique haplotypes were observed among Pacific Flyway populations (*n *= 84) characterized by 33 variable sites (Figure 2b). Nineteen of the 36 haplotypes (53%) were represented by a single individual (i.e., private), and only three haplotypes were shared among populations. Moderate‐to‐high levels of haplotype (*h* = 0.768–0.957) and nucleotide diversity (π = 0.0095–0.0215) were observed across populations, with the CIB and BBL populations having the lowest observed levels of genetic diversity (*p*s < .0001; Table 1).

**Table 1:** Estimates of genetic diversity of Pacific Flyway greater white‐fronted geese (*Anser albifrons*) including; average number of alleles, allelic richness (*r*), observed and expected heterozygosities (*H*_o_/*H*_e_), inbreeding coefficient (*F*_IS_), and sample size (*n*) calculated from eight microsatellite loci, as well as number of haplotypes, haplotype diversity (*h*), nucleotide diversity (π), Tajima's *D* and Fu's *F*, calculated from 365 bp of mtDNA control region

Population	Autosomal Microsatellites					mtDNA						
	*n*	No. Alleles	*r* a	*H*_o_/*H*_e_	*F*_IS_	*n*	No. Haplotypes	Polymorphic sites	*h* (SD)	π (SD)	Tajima *D*	Fu's *F*_s_
Bristol Bay	18	5.29	5.30	64.0/64.1	0.001	17	7	13	0.794 (0.078)	0.01067 (0.00630)	0.04	0.37
Cook Inlet	61	4.93	4.82	63.7/64.0	0.004	40	12	18	0.768 (0.060)	0.00948 (0.00549)	−0.58	−1.61
YK Delta	36	5.46	5.44	62.1/64.2	0.033	27	17	24	0.957 (0.021)	0.02154 (0.01152)	0.85	−3.42

a Allelic Richness (*r*) based on sample size of 15 from Bristol Bay, Alaska.

In 115 greater white‐fronted geese examined, the number of alleles per autosomal microsatellite locus ranged from 3 to 13, with an average of 7.0 alleles per locus. Molecular diversity indices were similar across populations with allelic richness (*r*) ranging from 4.8 to 5.4 (Table 1). Observed heterozygosity ranged from 58.4% to 61.4% for each population with an overall value of 59.6%. The inbreeding coefficient (*F*_IS_) ranged from 0.050 to 0.082 across sampled sites with an overall mean of 0.063; no *F*_IS_ value differed significantly from zero. All populations were in HWE, and no signature of linkage disequilibrium was detected between any pair of loci.

High genetic structure was observed at mtDNA control region (Φ_ST_ = 0.335, *p* < .001 and *F*_ST_ = 0.157, *p* < .001) with all pairwise comparisons showing significant differentiation (Table 2). Similarly, microsatellite loci exhibited a signature of population differentiation (*R*_ST_ = 0.130, *p* < .001 and *F*_ST_ = 0.020, *p* < .001). We estimated the upper limit of *F*_ST_ as 0.359 and 0.938 for *R*_ST_; thus, the standardized *F*_ST_ and *R*_ST_ is 0.056 and 0.139, respectively, which accounts for the maximum possible level of genetic structure based on our data set. In contrast to mtDNA sequence data, however, only the CIB (Tule) population was differentiated from the other populations based on autosomal loci (Table 2). In agreement, the STRUCTURE analysis, using sampling location as a prior (*r* > 1), suggested that the most likely number of populations is two, with the CIB population comprising one genetic cluster and other populations assigned to the second cluster (Figure 2c). The likely number of populations identified without locality priors, however, was *K* = 1.

**Table 2:** Pairwise and overall values of *F*_ST_, *R*_ST_, and Φ_ST_ calculated from eight microsatellite loci and 365 bp of mtDNA control region for Pacific Flyway populations of greater white‐fronted geese (*Anser albifrons*) in Alaska

	Autosomal Microsatellite		mtDNA	
	*F*_ST_	*R*_ST_	*F*_ST_	Φ_ST_
Bristol Bay				
Cook Inlet	**0.024**	**0.252**	**0.221**	**0.580**
YK Delta	0.000	0.003	**0.117**	**0.203**
Cook Inlet				
YK Delta	**0.024**	**0.119**	**0.134**	**0.217**
Overall	**0.020**	**0.130**	**0.157**	**0.335**

Numbers in bold are significant.

### 3.3 Recent and historical demography

All populations showed evidence of recent population decline or bottleneck (heterozygote excess; Table 3) based on the IAM. There was no evidence of significant heterozygosity excess or deficit in any of the populations under the SMM and TPM, indicating population equilibrium. Signatures based on mtDNA were consistent with long‐term population stasis and a lack of clear demographic expansion for all populations in the Pacific Flyway. In agreement, neither Tajima's *D* nor Fu's *F*_s_ were significantly negative for the mtDNA control region (Table 1), and the 95% confidence interval around the metric for population growth (*g*) overlapped or approached zero (LAMARC estimates are biased upward), consistent with a stable population size (Table 3).

**Table 3:** Analysis of historical fluctuations in population demography of greater white‐fronted geese (*Anser albifrons*) sampled in Alaska based on eight microsatellite loci using the infinite allele model (IAM), stepwise mutation model (SMM), and two‐phase model of mutation (TPM) and population‐growth‐rate parameter (*g*) for mtDNA control region

	Microsatellites			mtDNA	
	IAM	TPM	SMM	θ	*g* (95% CI)
Bristol Bay	Het exc (*p *= .00098)	Eq	Eq	0.012 (0.003–0.047)	54.4 (−292.7 to 717.9)
Cook Inlet	Het exc (*p *= .00293)	Eq	Eq	0.014 (0.006–0.034)	14.9 (−195.9 to 329.0)
YK Delta	Het exc (*p *= .00977)	Eq	Eq	0.084 (0.034–0.098)	197.5 (34.2–433.1)

### 3.4 Gene flow

**Figure 3:**
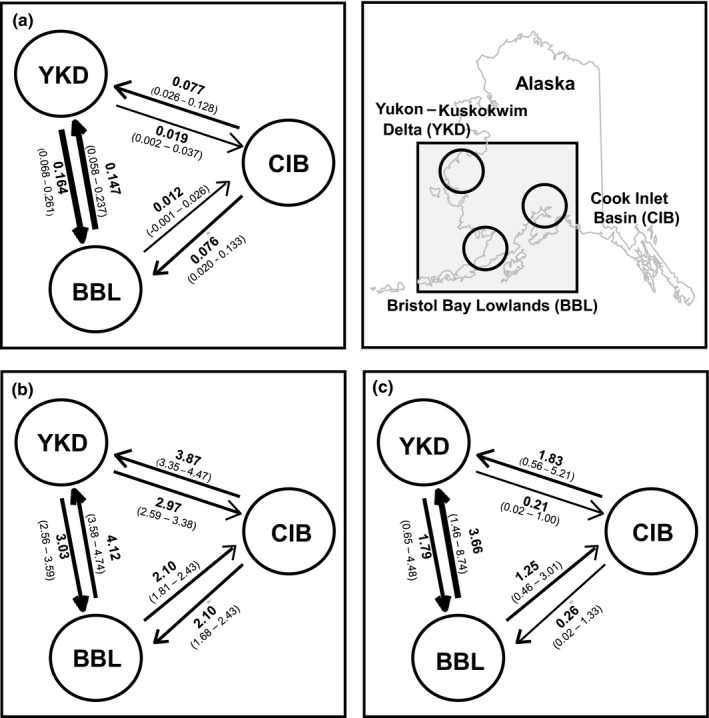
Rates of gene flow among three populations of greater white‐fronted geese (*Anser albifrons*) breeding in Alaska. (a) Contemporary gene flow (microsatellite analysis based on BayesAss); (b) Recent gene flow (microsatellite analysis using MIGRATE); and (c) Historical gene flow (analysis of mtDNA control region using MIGRATE). Numbers above arrows represent gene flow rates (proportion of individuals for BayesAss and number of migrants per generation for MIGRATE) in the direction of arrow. Numbers in brackets are 95% confidence intervals. Arrow thickness is scaled according to values

Although confidence intervals overlapped, there was weak asymmetrical contemporary and recent gene flow observed among sampled populations (Figure 3). On average, the directionality of gene flow was from either CIB or BBL into YKD based on mtDNA and microsatellite data. Under the BayesAss model for microsatellites, gene flow has been restricted into the CIB over the past several generations (i.e., contemporary gene flow), with approximately 3.1% of the population of migrant origin (Figure 3; panel a). This asymmetrical gene flow was also observed in MIGRATE for mtDNA, with generally more individuals emigrating from the CIB into the YKD than vice versa. In contrast to the restricted gene flow in and out of CIB, the BayesAss model indicated a higher rate of genetic exchange between BBL and YKD.

## 4 Discussion

We found strong differences in mtDNA haplotype frequencies among the three populations (Φ_ST_ = 0.355) with lower but significant differentiation in allelic frequencies involving CIB (*R*_ST_ = 0.130) which is concordant with morphological differences within the Pacific Flyway (Ely et al., 2005; Orthmeyer et al., 1995). Although a certain degree of differentiation is expected, as natal site fidelity is common in female waterfowl (Greenwood, 1980), the amount of genetic structure indicates that gene flow is at least partially impeded among these populations. Further, this divergence is in general not extremely recent (O'Reilly, Canino, Bailey, & Bentzen, 2004; Slatkin, 1995); *R*_ST_ values are larger than overall *F*_ST_ values, largely due to high *R*_ST_/*F*_ST_ ratios between CIB and the other populations (Table 2). This suggests that mutation is beginning to play a role in addition to dispersal and genetic drift in the differentiation between geese nesting in CIB, relative to the other populations. While it is possible that interdemic gene flow was initially restricted due to Pleistocene era isolation events (Ploeger, 1968), extant genetic structure in these seasonally sympatric populations is likely maintained by behavioral and ecological mechanisms affecting mate choice.

### 4.1 Isolating mechanisms: timing and location of mate choice

The degree of genetic structuring we found may be related to behavioral attributes which can influence population structure (Charpentier et al., 2012; Toews & Brelsford, 2012; Van Doornik, Berejikian, & Campbell, 2013). Mate selection in particular has a strong influence on gene flow, and many population genetics models assume that individuals in a population mate randomly, although this is not always true in natural populations (Bearhop et al., 2005; Thibert‐Plante & Gavrilets, 2013). Nonrandom mate selection can arise through a variety of passive and active mechanisms, which has been well studied in birds (Gowaty & Mock, 1985). Birds are predominantly socially monogamous (Lack, 1968), and northern‐breeding species generally pair and breed annually on nesting areas in spring or early summer. In long‐lived species with prolonged pair bonds such as geese, mate selection and reproduction may occur at different times and locations, which is especially likely in migratory species. For such species, knowledge of when and where pair formation takes place relative to the distribution of subunits of the population (i.e., proximity of potential mates) is more critical to understanding gene flow than knowing when actual breeding occurs. If mate selection is restricted to a specific time period in the annual cycle, the spatial distribution of potential mates during this “window” could have a profound effect on interdemic gene flow and eventual population structure. Individuals can be spatially segregated on a local scale by selecting sites which are favorable, and on a microscale by selecting specific habitats within a site. When spatial preferences act in concert with temporal differences in site use then even in the absence of active processes such as assortative mating, segments of a population may passively associate or disassociate, thereby greatly increasing the probability of nonrandom pairing. Cooke, Finney, and Rockwell (1976) referred to the effect of the relative availability of different phenotypes on mate choice as the “prevalence” hypothesis.

Although there have been few detailed studies on the timing of pairing in geese, it is apparent that there are differences among species in when individuals select mates (Ely & Scribner, 1994). These differences in turn can explain different patterns of genetic structure found among geese species. For example, genetic structuring in lesser snow goose (*Anser caerulescens caerulescens*) populations nesting on Wrangel Island, Russia (north and south wintering populations), and Banks Island, Canada (approximately 2,000 km apart), show a signature suggestive of winter pairing (Shorey et al., 2011). Populations that used common wintering sites (Banks Island and southern Wrangel Island) were more genetically similar than the two sympatric‐nesting Wrangel Island populations which winter allopatrically. Our data on greater white‐fronted geese show a reverse pattern, as pairing in greater white‐fronted geese occurs during spring or summer (Warren, Fox, Walsh, & O'Sullivan, 1992) when the populations in this study are separate. The two populations that were genetically the most distinct for both mtDNA and nuclear markers are sympatric during winter (YKD and CIB), but largely allopatric during spring and summer, whereas the genetically most similar populations (BBL and YKD) overlapped more in distribution during late winter and spring compared to CIB geese (Figure 2a; Ely & Takekawa, 1996; Ely, 2008) suggesting increased potential for interpopulation pairing. Population isolation during spring and summer pairing may also contribute to the high degree of population structuring observed in Canada geese (Scribner et al., 2003).

Despite the spatial overlap, Ely and Takekawa (1996) showed that YKD and BBL geese were temporally segregated during much of their annual cycle with BBL geese migrating earlier in spring and nesting earlier in Alaska than YKD geese. Ely and Takekawa (1996) suggested that the staggered breeding chronologies reduced the possibility of interpopulation pairing and likely impeded gene flow, which has subsequently been suggested as an isolating mechanism in other species (Bearhop et al., 2005; Friesen et al., 2007). CIB geese are similar to BBL geese in that they are also early spring migrants and early nesters (peak of hatch in early June versus late June for YKD geese); it is thus likely that they too are out of reproductive synchrony with YKD geese. Although breeding timing is similar between CIB and BBL geese, there is little if any overlap in spring migration pathways, and breeding areas are separated by >400 km. Our mtDNA results confirm a distinction among all three groups, although nuclear data show no significant differences between YKD and BBL geese, albeit only based on a limited number of nuclear markers.

### 4.2 Isolating mechanisms: ecological divergence

Reproductive isolation achieved through divergent selection on populations in contrasting environments can lead to speciation (Grant & Grant, 2006; McKinnon et al., 2004; Nosil, 2012; Ryan, Bloomer, Moloney, Grant, & Delport, 2007; Schluter, 2001, 2009; Stuart et al., 2014), and there is ample evidence to suggest that habitat segregation occurs among Pacific Flyway populations of greater white‐fronted geese. Differences in site preference is facilitated by the larger body size and robust bill of CIB geese which makes them better adapted than YKD or BBL geese for eating submerged aquatic marsh foods including the fibrous tubers of alkali bulrush (Ely, 2008). During winter, geese from the CIB (aka “Tule geese” or “timber goose based on the habitats it frequents; Swarth & Bryant, 1917) tend to remain in small, nonmixed flocks or segregated on the outer edge of flocks of YKD geese feeding on natural wetlands in the Sacramento Valley of California (Bauer, 1979; Delacour & Ripley, 1975; Hobbs, 1999). During spring, CIB geese feed primarily on submerged portions of aquatic plants in the Klamath Basin, and in southern Oregon (Ely, 2008; Wege, 1984). In contrast, YKD geese feed predominantly on agricultural crops during winter and spring (Ely & Raveling, 2011), as do BBL geese when they return to the Sacramento–San Joaquin Delta of California from Mexico in late winter (Ely & Raveling, 1989; Ely & Takekawa, 1996). Historically YKD and BBL geese in California likely fed on the seed heads and stalks of annual and perennial grasses, while the diet of CIB geese probably consisted of wetland plant species. Wetland losses in California, which have exceeded 90% since European settlement in the 1800s (Garone, 2011), have likely led to a decline in the potential for resource partitioning (and consequently increased gene flow) within greater white‐fronted geese. Differences among Pacific Flyway greater white‐fronted goose populations in site selection during spring and summer, when pair formation is thought to occur, is a corollary to the circumstance in three‐spine sticklebacks (*Gasterosteus aculeatus*) where two morphs feed in different habitats and also prefer different substrates for spawning, which further restricts gene flow (Schluter, 2009).

If body size is also an attribute under selection for pairing, as has been shown for Canada geese (MacInnes, 1966), and lesser snow geese (Ankney, 1977), then body size could act as a phenotypic characteristic under selection for both local adaptation and mate selection (i.e., a “magic trait”; Gavrilets, 2004; Servedio, 2000). The large body size and robust bill of CIB geese make them better adapted than YKD or BBL geese for eating submerged aquatic marsh foods including the fibrous tubers of alkali bulrush (Ely, 2008). Patten, Rotenberry, Zuk, and Shaw (2004) reported a similar situation for song sparrows whereby populations that foraged in denser habitats had different vocal characteristics, the latter of which influences mate choice. Such dual selection could explain the potentially rapid (post‐Pleistocene) divergence of Pacific Flyway populations of greater white‐fronted geese.

### 4.3 Behavior, culture, and imprinting

Our finding of significant population structuring within a single migratory flyway could be related to social drivers other than pair bonding behavior. Social characteristics, including prolonged family bonds, have been cited as a strong force in the genetic structuring of higher vertebrates, including killer whales (Foote, Newton, Piertney, Willerslev, & Gilbert, 2009; Hoelzel et al., 2007; Pilot, Dahlheim, & Hoelzel, 2010), and primates (Morin et al., 1994). Geese are similar to these species in that social aggregation and signaling is used to locate food. In geese, like other birds, flocking behavior facilitates food finding, while family dominance enhances food acquisition once at a feeding site (Boyd, 1953; Raveling, 1970). Also, site use and food preferences may be culturally transmitted in geese (Harrison et al., 2010), as young of many goose species remain with their parents throughout their first year of life, and in some species, including greater white‐fronted geese, offspring from previous years associate in extended family groups well into adulthood (Ely, 1993; Warren, Fox, Walsh, & O'Sullivan, 1993). The influence of family behavior on the population structure of geese was described by Mayr (1942), who attributed the pronounced races and inbreeding of small populations of Canada geese to both geographic isolation and family‐mediated social segregation. The importance of goose family behavior on genetic population structure was also reported by Cooke (1978) who showed that lesser snow geese select mates that have the same phenotypes as their siblings and parents.

Extended parental care can also affect population structure by providing added opportunity for offspring to imprint on parental phenotype. Sexual imprinting, whereby young birds learn species‐specific characteristics that inform mate selection (Irwin & Price, 1999), can play an important role in sympatric speciation (Higashi, Takimoto, & Yamamura, 1999; Kozak, Head, & Boughman, 2011). As sexual imprinting can influence mate choice later in life, and mate preferences can be transmitted across generations through cultural transmission, there is a mechanism for the evolution of mate preferences and restricted interdemic gene flow. Irwin and Price (1999) noted the importance of imprinting in speciation and concluded that the “role of behavior and learning in completing the speciation process is relatively overlooked.” The fact that sexual selection is operative in geese is supported by the evidence for assortative pairing in several species including lesser snow geese (Cooke, 1978; Cooke et al., 1976; Sutton, 1931), Canada geese (MacInnes, 1966), and brant (Abraham, Ankney, & Boyd, 1983). Winter site fidelity (e.g., Wilson, Norriss, Walsh, Fox, & Stroud, 1991), and year‐round associations of geese from the same breeding unit, as has been reported for Canada geese (Raveling, 1979; but not shown for greater snow geese—Desnoyers, Gauthier, & Lefebvre, 2012) may further restrict gene flow by effectively reducing the pool of individuals available as mates. Similarly, the temporal and spatial segregation we have documented among populations of greater white‐fronted geese increases the probability of young birds associating with other geese phenotypically (and genetically) similar to their parents, and eventually pairing with such individuals.

The behavior, cultures, and imprinting behavior of geese and swans (Tribe Anserini) is in contrast to most species of ducks (Oxyurini, Tadorinini, Aythinim, Anatini, Mergini; Anderson, Rhymer, & Rohwer, 1992) and may explain some of the overall differences in population structuring between the groups. Many different taxonomic groups within northern Anseriformes have similar dispersal potential, patterns of migration, and extant and historical geographic distribution (the latter being pertinent to vicariance events that can lead to speciation; Ploeger, 1968). Despite these similarities, the northern Anserini (geese and swans) are generally more polytypic than similarly distributed ducks, as 45% (9/20) of such Anserini species have recognized subspecies, compared to only 18% (9/49) of duck species (Appendix S1). Taxonomically classified polytypic subspecies are usually more genetically structured (Sonsthagen, Talbot, Scribner, & McCracken, 2011; Wagner & Baker, 1986). Even though female philopatry is common in most waterfowl species (Anderson et al., 1992; Greenwood, 1980), and many population genetic studies of ducks have employed maternally inherited mtDNA markers (which are expected to reveal more population structure than biparentally inherited markers, particularly when females are philopatric), most species of ducks show extensive population admixture (Kraus et al., 2011; Liu, Keller, & Heckel, 2012; Pearce et al., 2004; Wilson et al., 2016). This is true even of duck populations that have broad Holarctic distributions similar to the greater white‐fronted goose such as the northern Pintail (*Anas acuta*; Flint et al., 2010), and the mallard (*Anas platyrynchus*; Kraus et al., 2011). We propose that the more prominent genetic structuring of Northern Hemisphere geese and swans compared to ducks is due, at least in part, to differences in the degree of assortative pairing as influenced by timing of mate selection, social behavior (e.g., pair bond duration, degree of family structuring, and sexual imprinting) and, in some instances, morphologically mediated resource selection.

## 5 Conclusions

The moderate‐to‐high degree of genetic structuring and relatively low estimates of gene flow indicate the three populations of greater white‐fronted geese in the Pacific Flyway are at varying degrees of reproductive isolation and may be considered to be an example of early stage or incomplete radiation (whereby a lineage rapidly diversifies with emerging branches evolving different traits that may be adaptive; Givnish & Sytsma, 2000; Foote et al., 2009; Flohr, Blom, Rainey, & Beaumont, 2013). Gene flow among the three populations may be impeded by ecological partitioning and behavioral mechanisms such as long‐term pair bonds sexual imprinting, and a high degree of sociality which likely influence sexual selection. Many of these complex behavioral attributes are specific to geese and swans, and not ducks generally (Anderson et al., 1992). Seventy years ago, Wright (1946), in a paper addressing the influence of behavior on population structure, coined the term “neighborhood” to define a situation when the location of potential parents is not random, but correlated. For migratory birds with strong site fidelity, such neighborhoods are mobile and could in fact be considered “caravans.” For species with multiple drivers impeding gene flow such as greater white‐fronted geese, such neighborhoods and caravans may be “exclusive.”

## Conflict of Interest

None declared.
